# Molecular Modeling for Nanomaterial–Biology Interactions: Opportunities, Challenges, and Perspectives

**DOI:** 10.3389/fbioe.2019.00268

**Published:** 2019-10-17

**Authors:** Tommaso Casalini, Vittorio Limongelli, Mélanie Schmutz, Claudia Som, Olivier Jordan, Peter Wick, Gerrit Borchard, Giuseppe Perale

**Affiliations:** ^1^Polymer Engineering Laboratory, Department of Innovative Technologies, Institute for Mechanical Engineering and Materials Technology, University of Applied Sciences and Arts of Southern Switzerland (SUPSI), Manno, Switzerland; ^2^Faculty of Biomedical Sciences, Center for Computational Medicine in Cardiology, Institute of Computational Science, Università della Svizzera italiana (USI), Lugano, Switzerland; ^3^Department of Pharmacy, University of Naples “Federico II”, Naples, Italy; ^4^Technology and Society Laboratory, Swiss Federal Laboratories for Materials Science and Technology (Empa), St. Gallen, Switzerland; ^5^School of Pharmaceutical Sciences, University of Geneva, Genève, Switzerland; ^6^Laboratory for Particles – Biology Interactions, Swiss Federal Laboratories for Materials Science and Technology (Empa), St. Gallen, Switzerland; ^7^Ludwig Boltzmann Institute for Experimental and Clinical Traumatology, Wien, Austria

**Keywords:** molecular dynamics, metadynamics, molecular modeling, protein corona, coarse grain, lipid bilayer, cellular membrane

## Abstract

Injection of nanoparticles (NP) into the bloodstream leads to the formation of a so-called “nano–bio” interface where dynamic interactions between nanoparticle surfaces and blood components take place. A common consequence is the formation of the protein corona, that is, a network of adsorbed proteins that can strongly alter the surface properties of the nanoparticle. The protein corona and the resulting structural changes experienced by adsorbed proteins can lead to substantial deviations from the expected cellular uptake as well as biological responses such as NP aggregation and NP-induced protein fibrillation, NP interference with enzymatic activity, or the exposure of new antigenic epitopes. Achieving a detailed understanding of the nano–bio interface is still challenging due to the synergistic effects of several influencing factors like pH, ionic strength, and hydrophobic effects, to name just a few. Because of the multiscale complexity of the system, modeling approaches at a molecular level represent the ideal choice for a detailed understanding of the driving forces and, in particular, the early events at the nano–bio interface. This review aims at exploring and discussing the opportunities and perspectives offered by molecular modeling in this field through selected examples from literature.

## Introduction

Nanomedicine is an emerging discipline that is providing novel impulses to the biomedical field thanks to the use of nanotechnologies and the continuous development of engineered nanomaterials such as polymer-, metal- or metal oxide-based nanoparticles. Nanomaterials, by virtue of their small size (1–1000 nm, comparable to many biological molecules like proteins and viruses) open up a wide range of new opportunities and applications, for example as devices for targeted drug delivery and diagnostic purposes and as image contrast agents. However, as with every novel technology, the potential negative side effects have to be assessed early in the development process to avoid adverse social and economic effects.

Indeed, the injection of nanomaterials into an organism leads to complex interactions between the surface of the device and the components of the medium, such as proteins, carbohydrates, fatty acids, *et cetera*. These interactions play a key role in determining not only the fate of the nanomaterial (in terms of clearance and *in vivo* biodistribution) but also the attainment of undesired side effects. The fundamental driving forces governing the formation of this nano-bio interface have already been identified and discussed (Nel et al., 2009) and include van der Waals and electrostatic interactions and hydrophobic and depletion effects. The challenge lies in the rationalization of the synergistic effects of intrinsic nanomaterial properties (chemical composition, size, surface functionalization, *et cetera*), the characteristics of the surrounding medium (pH, ionic strength, *et cetera*), and the phenomena occurring at the interface and their impact on nanomaterial behavior.

One of the most relevant consequences is the formation of the protein corona, i.e., a layer of adsorbed proteins on the NP surface (Cedervall et al., 2007a,b; Lundqvist et al., 2008; Dell'orco et al., 2010). The attainment of such a network alters the surface properties of the nanomaterial, which may cause substantial deviations from the expected behavior concerning colloidal stability, cellular uptake, clearance, distribution within the organs, and immune response.

On top of that, the formation of the protein corona can lead to changes in the protein structure and thus to undesired consequences (not easily predictable *a priori*), such as (Nel et al., 2009):

- Enhanced or hampered cellular uptake with specific kinds of cells due to the interactions of adsorbed proteins with particular receptors;
- Protein aggregation and fibrillation at the nanocarrier surface;
- Interference with enzymatic activity;
- Exposure of new antigenic epitopes.

Experimental protocols for the investigation of the protein corona are currently well-established (Walkey and Chan, 2012; Wei et al., 2014; Pederzoli et al., 2017), although they have some intrinsic limitations concerning spatial and temporal resolution; indeed, they do not allow the characterization of the early events leading to protein corona formation and do not provide a clear overview of specific nanomaterial/protein interactions or changes in protein structure.

Computational approaches at the molecular scale, such as molecular dynamics (MD) simulations, constitute the natural complement to experimental techniques. This is due to several factors, such as the accessible time and length scales (microsecond and nanometer, respectively), the full atomistic description of the system (which allows the specific protein/nanomaterial interactions to be identified) and its dynamic behavior (thus identifying conformational changes after binding), and the inclusion of environmental effects.

This review aims at exploring and discussing the opportunities and limitations of nano-bio as well as giving some perspectives on the use of molecular modeling techniques for characterizing these interactions. After giving a brief theoretical background, relevant applications of simulations at the molecular scale are discussed through selected examples from the scientific literature.

## Molecular modeling—a brief overview

Molecular modeling can be seen as the sum of two components: a molecular model and a computational technique to properly characterize the behavior of the molecules.

Building a suitable molecular model, that is, how the system under investigation is rationalized and represented in the framework of a meaningful simulation, is the first fundamental step. In this framework, molecular models can be essentially divided into two categories; on the one side, full atomistic models provide the highest level of detail since all atoms (considered as the smallest constitutive units of the model) are explicitly accounted for. On the other side, coarse-grained models summarize the atomic detail by enclosing groups of atoms into beads that lump the main peculiarities (in terms of charge, polarity, *et cetera*) of the atoms that they embed. This simplification is unavoidable for complex systems whose atomistic representation would be prohibitive from a computational point of view, in terms of the system size and/or time and length scales needed to investigate the phenomena of interest. Despite the loss of detail, a coarse-grained model that retains the main features of the system is able to provide meaningful insights at a reasonable computational cost (*vide infra*). For the sake of completeness, there exist more detailed representations where electrons are the smallest constitutive units and are explicitly included. Such models are treated with quantum chemistry methods, which are not considered or discussed here since their application in the field of nanomedicine is hindered by their computational inefficiency.

In a broader sense, a molecular model also includes unavoidable simplifications that allow for the simulation of complex systems, either at a full atomistic or coarse-grained level of detail, which could not be treated otherwise. The simulation of protein adsorption on a microparticle surface, for example, is unfeasible because of the system size. Such a system is usually simplified by adopting a molecular model that involves the adsorption of a protein on a flat surface with a suitable thickness. This approach is reasonable since the phenomena of interest are restricted to the solvent/particle interface; in addition, since protein size is much smaller than microparticle radius, curvature effects can be reasonably neglected.

The second component of molecular modeling is constituted by suitable computational methods that allow the characterization of the dynamics, energetics, and conformational sampling of the system of interest. Full atomistic models are usually treated with molecular dynamics, while other techniques such as coarse-grained molecular dynamics and dissipative particle dynamics are employed along with coarse-grained models.

Each method has its own strengths and limitations, as well as characteristic accessible time and length scales, as discussed in the following paragraphs.

### Full Atomistic Models—Molecular Dynamics

In molecular dynamics simulations, atoms are represented as spheres that interact with each other by virtue of a potential energy function, usually called the force field (FF). Molecular coordinates and velocities as a function of simulation time can be evaluated by solving Newton's equation of motion with a suitable numerical integration scheme, as shown in Equation (1) (Frenkel and Smit, 2002):

(1) $$\begin{array}{r} m_{i}\frac{d^{2}r_{i}}{dt^{2}}=F_{i}=-\nabla U\left(r\right) \end{array}$$

where *m*_*i*_ is the mass of the i-th atom, *r*_*i*_ are the spatial coordinates of the i-th atom, *t* is time, *F*_*i*_ is the force acting on the i-th atom, and *U(r)* is the potential energy (that is, the force field), which is a function of the coordinates of all atoms present in system *r*. Such an approach essentially implies a couple of assumptions, as follows. First, the motion of electrons can be reasonably described by the dynamics of the corresponding nuclei (Born–Oppenheimer approximation). Second, the motion of the atomic nuclei (which are heavier than electrons) can be described as point particles that follow classical mechanics; this is an acceptable approximation when quantum effects are not important (Frenkel and Smit, 2002). Generally speaking, a force field takes into account both intramolecular and intermolecular interactions, in terms of bonds, angles, dihedrals, and long-range interactions, namely van der Waals and electrostatic.

FFs contain several parameters that are computed in order to reproduce the conformational energies and minimum energy structures obtained from high-level quantum mechanics calculations and/or experimental data, such as hydration enthalpies or structural parameters from NMR experiments (Riniker, 2018). There are “general purpose” force fields, usually employed to describe small ligands, as well as FFs specifically tailored for given categories of molecules, like proteins, nucleic acids, carbohydrates, and lipids (Riniker, 2018). The choice and the quality of the force field cannot be underestimated, since they strongly affect the reliability of the simulation outcome.

MD simulations do not explicitly consider electrons, so chemical reactions and excited states cannot be investigated; however, they constitute the ideal tool for those systems that are mainly governed by non-covalent interactions, like electrostatic and Van der Waals forces. MD also allows environmental conditions to be included through the addition of explicit solvent molecules, ions, and other solute molecules into the system. The main outputs from an MD simulation are molecular trajectories, the post-processing of which can provide structural information (binding poses, protein conformation) as well as energetic information such as interaction energies.

### Enhanced Sampling Methods

The characteristic time and length scales of MD simulations are in the tens to hundreds of nanoseconds (up to 1000 ns) and tens of nanometers (up to 20 nm), respectively. However, many phenomena of interest (e.g., molecular binding, protein unfolding) need large time scales to occur (up to minutes), and their investigation through MD would be in principle unfeasible; this is due to the presence of metastable states separated by high free energy barriers. A way to overcome this issue is to use enhanced sampling methods, which allow enhancement of the transitions between different metastable states separated by energy barriers higher than the thermal energy *k*_*B*_*T*, which would not be crossed in a standard simulation at temperature *T* (where *k*_*B*_ is the Boltzmann constant and *T* is absolute temperature). As recently reviewed (Camilloni and Pietrucci, 2018), there are three different suitable approaches: i) increasing the temperature *T*; ii) changing the potential *U(r)*, and iii) adding an external bias potential *V(r)*. Each approach has its own methods, the discussion of which (along with their theoretical basis) is well beyond the purpose of this review; the interested reader is referred to *ad hoc* reviews (Miao and Mccammon, 2016; Camilloni and Pietrucci, 2018). Some of the popular enhanced sampling techniques are Replica Exchange (RE, first approach) (Miao and Mccammon, 2016) and Well-Tempered Metadynamics (WTM) (Valsson et al., 2016), which belongs to the third group. In particular, WTM and its variant forms allow the free energy of the system under investigation to be recovered by adding an external bias on a selected number of degrees of freedom, commonly referred to as collective variables (CVs). CVs are generally functions of atomic coordinates and can range from simple quantities, such as distances and dihedral angles, to more complicated variables, like the number of hydrogen bonds/hydrophobic contacts, alpha helix-content in a protein, or Debye–Hückel interaction energy. Collective variables must be chosen so that they can discriminate between metastable states and can be representative of the transition mechanism. Typical applications of WTM and WTM-based methods are the study of protein conformations (also in the presence of denaturants) (Owczarz et al., 2015), the binding poses of small ligands to target proteins (Tiwary et al., 2015), and the conformation and self-assembly of polymeric and supramolecular systems (Bochicchio and Pavan, 2018). Some phenomena, such as protein folding, require a relevant number of collective variables to perform meaningful simulations. Although conceptually feasible, running a WTM simulation with many CVs introduces some issues such as a drop in computational efficiency and a non–trivial analysis of the results obtained. In order to overcome this issue, some WTM variants have been proposed, discussed, and validated in literature (mainly for protein folding), namely Parallel Tempering Metadynamics (PTMD) (Bussi et al., 2006), Parallel Tempering Metadynamics in the Well-Tempered Ensemble (PTMD-WTE) (Deighan et al., 2012), and Bias Exchange Metadynamics (BEMD) (Piana and Laio, 2007). The discussion of the theoretical basis of these methods is beyond the purpose of this review; the interested reader is referred to the corresponding papers.

### Coarse-Grained Models—Molecular Dynamics, Dissipative Particle Dynamics

The aim of coarse-grained (CG) models is to perform meaningful simulations of systems whose analysis would be challenging or unfeasible with full atomistic MD methods by building simplified representations that allow the main physical/chemical features (like the interplay between hydrophobic and hydrophilic effects) to be retained.

In the coarse-graining procedure, groups of atoms are enclosed into “beads” or “interaction sites” that are representative of the embedded atoms in terms of charge, size, hydrophobicity/hydrophilicity, *et cetera*. Beads interact with each other by virtue of a potential energy function, which takes into account both bonded interactions (that is, bond, angles, and dihedrals) and non-bonded interactions and which is parameterized in order to optimally reproduce some experimental properties (like water/octanol partition) or the behavior of more detailed full atomistic simulations.

Trajectories can be computed by integrating Newton's equation of motion and also adding other components to the force such as friction due to the solvent (if implicit solvent methods are used) (*vide infra*).

It is worth mentioning that the coarse-graining procedure can be performed to different extents, since a bead can enclose a group of atoms (3–4 heavy atoms), a group of monomers (or amino acids), an entire protein or an entire microparticle, according to the aim of the simulation. In this review, the term “coarse-grained models” is employed for all those approaches where there is a loss of degrees of freedom with respect to a full atomistic description.

**Figure 1:**
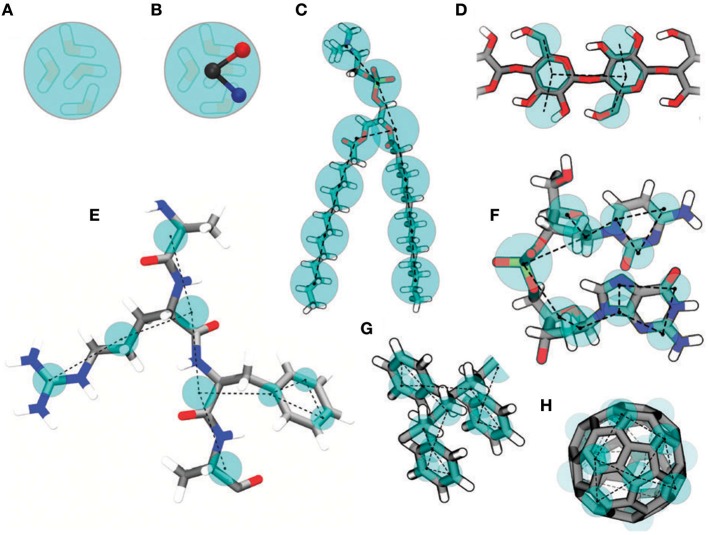
Examples of MARTINI mapping. Standard water bead embedding four water molecules **(A)**. Polarizable water bead with embedded charges **(B)**. DMPC lipid **(C)**. Polysaccharide fragment **(D)**. Peptide **(E)**. DNA fragment **(F)**. Polystyrene fragment **(G)**. Fullerene **(H)**. Reproduced from Marrink and Tieleman (2013) under a CC-BY 3.0 license. Published by the Royal Society of Chemistry.

A common drawback of CG models is that parameterization is strictly tailored for the system under investigation and in principle should be repeated for every new system; in other words, parameters are not transferable. In this regard, the MARTINI force field (Marrink et al., 2007) attracted a lot of interest due to its reliability and straightforward coarse-graining procedure. Beads (which include groups of 3–4 heavy atoms) still interact with each other through a simple potential energy function, as described for MD (*vide supra*). MARTINI offers a library of parameterized beads, mainly divided into four categories: polar, non-polar, apolar, and charged; in addition, each group includes subgroups representative of polarity and hydrogen bond capability. Parameters for bonded interactions (bonds, angle, dihedrals) must be determined from detailed MD simulations, while non-bonded interactions are tuned in order to reproduce thermodynamic properties like free energy of hydration, free energy of vaporization, and partitioning between water and different solvents. Explicit water and ions can also be added (a MARTINI water bead is representative of four water molecules). An example of MARTINI mapping from a full atomistic to a coarse-grained system is shown in Figure 1.

Bead parameterization can be further refined by the user in order to improve agreement with full atomistic simulations. Even with simulations based on the MARTINI force field, some phenomena of interest can be still characterized at a time scale that is not accessible. In this framework, enhanced sampling methods like Metadynamics can be employed to alleviate this issue, as already shown in the literature (Lelimousin et al., 2016).

Another widely employed method with CG models is Dissipative Particle Dynamics (DPD). Bead trajectories are still obtained by means of Newton's equation of motion, assuming that each i-th particle is subjected to three pair-additive forces that arise from the interactions with the other j-th particles: a conservative force, a dissipative force, and a random force (Liu et al., 2015):

(2) $$\begin{array}{r} m_{i}\frac{d^{2}r_{i}}{dt^{2}}=f_{i}=\sum_{j\neq i}F_{ij}^{c}+F_{ij}^{d}+F_{ij}^{r} \end{array}$$

The conservative force *F*^*c*^ is due to the interaction potential of particles and accounts for both bonded and long-range interactions through an elastic force and a soft repulsion force, respectively. *F*^*d*^ is a dissipative force that damps the relative motion between particles, and *F*^*r*^ is a random force directed along the line that connects beads centers. Dissipative and random forces are momentum-conserving and represent the minimal model that takes into account viscous forces and thermal noise between particles.

### Full Atomistic vs. Coarse-Grained Models: Strengths and Weaknesses for Nanomaterial–Biology Interactions

In this framework, full atomistic models provide the highest level of detail, since all atoms are explicitly included. On the one side, they account for all those fundamental interactions that are essential for a suitable description of the nano–bio interface, such as van der Waals, electrostatic, hydrogen bonding, π-π stacking, and π – cation interactions (provided the intrinsic limits and the accuracy of the FF). On the other side, the inclusion of explicit solvent molecules, ions, and other solute molecules allows environmental effects to be taken into account; the impact of pH is accounted for by appropriately changing protonation states. Focusing on proteins, by means of molecular dynamics simulations and their resolution at atomic scale it is possible to highlight the most relevant amino acids that drive the interactions at the nano–bio interface and protein structural changes at the single amino acid level, achieving a level of detail that is usually out of reach from an experimental point of view. On top of this, the reliability of the simulation results can be assessed by comparing theoretical quantities such as circular dichroism spectra with the corresponding experimental outcomes. The importance of this aspect cannot be underestimated since it strengthens the connection between experiments and simulations. Molecular dynamics simulations are still limited by the characteristic time and length scales accessible by the method: microseconds and nanometers, respectively. The direct use of enhanced sampling methods is still prohibitive for complex and/or large systems. In this regard, switching to coarse-grained models is a forced but attractive choice due to the longer accessible time and length scales (tens of microseconds and tens of nanometers, respectively). The drawback is the loss of the atomic detail, which implies that some interactions (strong electrostatic interactions, hydrogen bonds, solvation effects) are accounted for only in a roughly qualitative way. Anyway, if the fundamental physical/chemical peculiarities of the system (such as the balance between hydrophobic and hydrophilic groups) are well reproduced in the CG model and if the interaction potentials (that govern the interactions between beads) are accurately parameterized against experimental data or validated simulations at atomic scale, simulations at CG scale are a powerful tool to complement the insights obtained with MD simulations. CG simulations can also provide some input guess structures for, e.g., protein–protein interactions (that would be challenging to obtain with MD simulations), which can be further employed for a more accurate analysis at atomic scale. On top of that, enhanced sampling methods (in particular, Well-Tempered Metadynamics) have proved to be useful for simulations at CG scale when the time scale is still not accessible.

All these aspects are discussed in detail, along with selected examples, in the following paragraphs.

## Applications for nanomaterial–biology interactions

Molecular modeling is essentially employed for two purposes in the framework of nanomaterial–biology interactions. On the one side, it can shed light on the early events leading to the protein corona, highlighting the main mechanisms behind protein adsorption on the nanomaterial surface (hydrophobic effects, hydrogen bonds, electrostatic interactions, *et cetera*), the most important amino acids involved in the binding and the attainment of conformational changes. On the other side, simulations at the molecular scale allow the evaluation (in a trend-wise manner) of the impact of environmental effects, nanoparticle material, and surface functionalization on cellular uptake; some preliminary theoretical insights can also be obtained concerning the effect of protein corona formation.

### Protein Corona

Molecular modeling, thanks to its resolution at the atomic scale, represents the natural choice for the study of early events that lead to protein corona formation. Knowledge of the structural changes experienced by the protein after adsorption is essential for understanding system behavior, as discussed in the introduction (*vide supra*). Molecular modeling can offer an exhaustive overview of the structural transitions thanks to the resolution at a molecular level, highlighting the portion of proteins subjected to structural changes (along with the most important amino acids that cause this) and the main driving forces (electrostatic interactions, hydrophobic effects, *et cetera*). This allows information to be obtained that is challenging or impossible to achieve experimentally, and this is why molecular modeling has emerged as the natural and ideal complement to experiments. A typical application is constituted by detailed MD simulations of the interactions between a protein and a particle and the resulting changes in protein structure. The particle is usually modeled as a flat surface. On the one hand, there is no need to account for the entire sphere, since the interactions occur only at the interface. On the other hand, if the size of the protein is much smaller than the particle size, surface curvature effects can be safely neglected; this approximation is not valid for nanoparticles, whose size is comparable to those of proteins, and particle curvature must be accounted for by building the molecular model of the NP surface properly.

In this framework, full atomistic simulations can provide a detailed picture of the structural changes experienced by the protein after adsorption at the surface in terms of modifications of its secondary and tertiary structure (increase/decrease of alpha-helix and beta-sheet motifs and their arrangement). The specific structural changes of the protein can be directly correlated with experimental data, circular dichroism results, or NMR spectra. In addition, since protein adsorption modifies the properties of the particle surface (in terms of charge, hydrophobicity, *et cetera*), the insights obtained can be correlated, e.g., to differences in the colloidal stability of the particle suspension or other phenomena related to the protein corona such as protein aggregation and fibrillation.

Environmental effects can be taken into account thanks to the addition of explicit solvent molecules and ions, so that given salt concentrations (i.e., ionic strength) can be included in the simulation. The effect of pH can be included by changing the protonation state of the protein and the NP surface accordingly; anyway, protonation states in MD simulations are fixed and not dynamic since proton exchanges are not simulated. In other words, a positively charged amino acid remains protonated during the entire simulation, although the proton may be exchanged with surrounding water molecules according to the environmental pH. On top of that, the acid dissociation constant can be heavily influenced by local environmental effects such as the neighboring units and exposure to the solvent. This issue can be overcome by means of constant pH methods, which are currently available and validated only for proteins (Swails et al., 2014).

Simulations can also account for surface functionalization and its impact on the interactions with the protein. Through trajectory post-processing, it is possible to identify the main driving forces behind adsorption (hydrophobic effects, hydrogen bonds, *et cetera*) and to compute interaction energies in order to obtain a quantitative estimation of the strength of the binding.

Although the results of such simulations can surely contribute to increasing understanding and rationalizing experimental data, this approach also has some limitations and drawbacks.

The accuracy and reliability of the simulated protein structural changes are strongly related to the robustness of the force field; if FF parameterization leads to, e.g., an overestimation of alpha-helix content, this will unavoidably affect the simulation results. Several articles where force field performances are systematically analyzed, as well as reference FF papers, address such limitations in detail, which are therefore known *a priori*. It is also worth mentioning that force field improvements are continuously carried out, and updated FF versions are periodically released. In principle, changes in protein secondary and tertiary structure can occur on time scales beyond those accessible to standard MD simulations (ns–μs), so the use of enhanced sampling methods often becomes an inescapable necessity to achieve meaningful results. Standard MD simulations provide an ensemble of conformations according to the given conditions (temperature, solvent, ionic strength, *et cetera*), but if two metastable states are separated by an energy barrier much higher than the thermal energy, *k*_*B*_*T*, some relevant protein conformations are not accounted for because this barrier would not be crossed and simulation outcomes can provide only a partial description of the event under investigation. The use of enhanced sampling methods alleviates this issue.

Simulations are usually focused on the adsorption of a single protein on a surface, which is essentially representative of particles in a very dilute protein solution; in other words, the overall protein–protein interactions are neglected since they can occur on long time scales and their description is usually challenging, even with enhanced sampling methods. Although simulations provide interesting insights, systematic and rational validation of the molecular models is still lacking. This currently hinders the extensive use of molecular simulations for practical applications, such as the engineering of nanoparticles in order to promote or discourage the adsorption of given proteins.

In this regard, the use of coarse-grained models, along with suitable techniques to study system dynamics, represents an inescapable choice, although the atomic detail is lost. CG models allow longer time and length scales to be explored than do full atomistic models coupled with MD simulations and can thus be used to investigate the impact of protein–protein interactions, overcoming the infinite dilution condition. Small nanoparticles can be explicitly included, and the surface curvature effect can be taken into account. However, the coarse-graining procedure is not painless due to its intrinsic limits: strong electrostatic interactions, solvation effects, and anisotropic interactions like hydrogen bonding are poorly described. Focusing on proteins, it is still challenging to account for changes in secondary structures. Therefore, an accurate parameterization of interaction potentials is an essential step in obtaining reliable results. Simulations at CG scale, despite the mentioned drawbacks, can still provide useful insights and can also be employed to obtain input guess structures for protein–protein interactions that can subsequently be investigated at an atomic level. The interaction potentials are usually parameterized against more accurate simulations with full atomistic models, whose validity, in turn, must be evaluated through comparison with experimental data. This further reinforces the need for systematic experimental validation.

The advantages and disadvantages (related to both MD and CG approaches) are summarized in Table 1.

**Table 1:** Advantages and disadvantages in protein–surface simulation.

**Advantages**	**Disadvantages**
Detailed overview of protein structural changes after adsorption at single amino acid level	Intrinsic limits due to the accuracy of the employed force field
Explicit solvent molecules and ions allow environmental effects to be accounted for	Standard simulation may not be sufficient to account for protein structural changes due to time scale limitations; results from enhanced sampling methods still heavily depend on FF accuracy, which must be assessed with experiments
pH effects through protonation state of protein and surface	Protein–protein interactions are usually neglected; they can be accounted for with CG models, but systematic model validation is still lacking
Impact of particle material and surface functionalization on protein structure and adsorption	Lack of systematic validation through comparison with experimental data

As mentioned above, molecular models still need to be validated against comparison with experimental data. Indeed, for every property of interest, it is possible to highlight reference experimental techniques as well as computational techniques, as summarized in Table 2.

**Table 2:** Reference experimental and computational techniques for properties of interest of the protein corona.

**Property of interest**	**Experimental technique**	**Computational technique**
Particle stability	Dynamic light scattering, zeta potential	Assessment of surface hydrophilicity/hydrophobicity changes upon protein adsorption
Protein conformational changes	Circular dichroism, nuclear magnetic resonance	Standard molecular dynamics simulations and enhanced sampling methods provide insights into conformational changes at single amino acid level Theoretical circular dichroism spectra can be obtained from simulations
Adsorption thermodynamics	Isothermal titration calorimetry	Protein–surface interaction energy or binding free energy from post-processing of molecular trajectory; binding free energy from enhanced sampling methods

The literature offers several examples of MD simulations of protein adsorption on different materials, such as graphene sheets (Chong et al., 2015), carbon nanotubes (Ge et al., 2011; Gu et al., 2015), gold nanoparticles/surfaces (Wang et al., 2013; Brancolini et al., 2014; Tavanti et al., 2015; Bellucci et al., 2016; Yang et al., 2017; Ma et al., 2018), hydroxyapatite surfaces (Dong et al., 2007, 2011), fullerenes (Leonis et al., 2015), titanium oxide surfaces (Utesch et al., 2011; Mudunkotuwa and Grassian, 2014), and molybdenum disulfide (Gu et al., 2018), highlighting the specific interactions behind the binding and the attainment of structural changes. Interestingly, there are no relevant computational studies of protein adsorption on polymer surfaces. To our best knowledge, this may be due to the limited availability of validated FF parameters for polymers and to intrinsic issues with the design of molecular models. Whereas inorganic nanoparticles are characterized by an ordered atomic arrangement, a model of a disordered polymer random coil can be more challenging to build.

Among many theoretical works, only a few papers combine experimental and computational components in order to achieve an all-round understanding of the mechanisms that lead to hard corona formation. Chong et al. (2015) adopted MD simulations to study the affinity of four abundant plasma proteins (bovine fibrinogen, immunoglobulin, transferrin, and bovine serum albumin) on graphene oxide and reduced graphene oxide surfaces. The affinity trend predicted by MD is in agreement with the experimental trend for all investigated proteins. Simulations also allowed determination of the most relevant residues for the binding. Gu et al. (2018) studied the interactions of MoS_2_ nanoflakes with potassium channels proteins highlighting potential toxic effects of the binding, which can alter the biological function. The results were further corroborated by experimental data.

As mentioned, enhanced sampling methods are currently also applied for the study of protein–surface interactions with both MD simulations (where the system is described at full atomistic level) and CG simulations (where the atomic detail is lost for the sake of computational efficiency). Indeed, the accessible time scale may not be adequate for the phenomena under investigation, and the use of enhanced sampling methods is a good solution for both MD and CG simulations.

**Figure 2:**
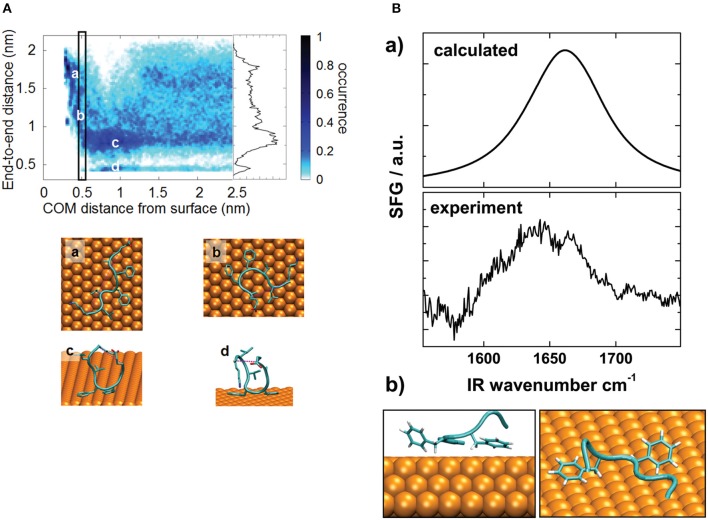
**(A)** Distribution fraction of peptide end-to-end distance (computed considering terminal C_α_ atoms) as a function of peptide–surface distance. The rectangle identifies the free energy minimum as a function of the peptide–surface distance. The inset represents the distribution of the end-to-end distance in the bulk region (COM distance from the surface larger than 1.25 nm). (a–d) show representative conformations. **(B)** Comparison between calculated and experimental SFG spectra (a) and simulated structure used for spectral calculation (b). Reproduced from Bellucci et al. (2016) under a CC-BY 3.0 license. Published by the Royal Society of Chemistry.

Even if standard simulations are sufficient for small peptides, the application of enhanced sampling methods improves the efficiency of the sampling and provides additional information about the system thanks to the possibility of reconstructing the free energy as a function of the degrees of freedom of interest. In this regard, Metadynamics-based methods have proved to be a promising choice. Prakash et al. (2018) systematically analyzed the use of Metadynamics-based methods for the adsorption of GGKGG peptide on a silica surface, explicitly including the influence of ionic strength and ion charge; the authors discussed the performances of each method and suggested the best collective variables to account for, thus providing useful guidelines for meaningful simulations. Deighan and Pfaendtner (2013) employed Metadynamics to study the influence of surface functionalization on the adsorption of Lkα14 and Lkβ15 peptides on self-assembled monolayers; the model outcomes were in good agreement with experimental findings. Bellucci et al. (2016) investigated the adsorption of Aβ_16−22_ peptide on a gold surface in order to investigate the impact of the binding on fibrillation. Their simulations revealed that binding poses are mainly influenced by the affinity between gold and phenylalanine, as shown in Figure 2A. The model was also validated through a comparison between experimental and calculated spectra obtained through sum generation frequency (SFG) spectroscopy (Figure 2B).

Hildebrand et al. (2018) employed Metadynamics-based methods to examine the conformational changes of Chymotrypsin after adsorption on silica. Simulations highlighted that the enzyme loses part of its helical content with minor perturbation of the tertiary structure; the model results were used to compute a theoretical circular dichroism spectrum that was in good agreement with the experimental spectrum.

CG models are also extensively used (Bellion et al., 2008; Vilaseca et al., 2013; Ding and Ma, 2014; Lopez and Lobaskin, 2015; Tavanti et al., 2015; Yu and Zhou, 2016; Hu et al., 2017; Wei et al., 2017), since they allow the characteristic accessible time and length scales of full atomistic simulations to be extended and the computational cost to be reduced. It thus becomes possible to simulate entire nanoparticles whose size is equal to or less than about 20 nm (at least when MARTINI is employed), fully covered by one or more kinds of proteins. The investigation of larger particles is still also challenging for CG methods because of the required computational effort.

Adopting CG models implies the loss of the atomic detail at the single amino acid level and a less accurate description of the system. While hydrophobic effects are reasonably accounted for, it is challenging to take into account properly, e.g., water structuring, cation–π interactions, strong electrostatic interactions, and hydrogen bonds, which lose their directionality because of the coarse-graining procedure (Marrink and Tieleman, 2013). Focusing on proteins, changes in tertiary structure can be reasonably described, while it is still non-trivial to account for changes in secondary structure due to the intrinsic limitations of the method (Marrink and Tieleman, 2013).

Despite such limitations, CG models can be employed for qualitative insights or to obtain guess structures for subsequent more detailed full atomistic simulations, as is commonly done, e.g., for the non-covalent protein–protein interaction and oligomerization of membrane proteins (Lelimousin et al., 2016). Anyway, a systematic use for more quantitative results must first be corroborated through comparison with more accurate and, above all, validated atomistic MD simulations.

Yu and Zhou (2016) used CG simulations with the MARTINI force field to understand the influence of nanoparticle curvature on lysozyme adsorption on silica at different values of ionic strength. They found that while salt concentration had a modest effect, surface curvature greatly influenced structural changes.

Ding and Ma (2014) used dissipative particle dynamics to characterize the adsorption of human serum albumin (HSA) on generic hydrophobic, hydrophilic, and charged nanoparticles for different size and pH values. By computing binding free energy as a function of the distance between the protein and particle centers of mass (COM), they showed that HSA could be bound only to hydrophobic and positively charged nanoparticles. They further studied the attainment of the protein corona by computing the number of adsorbed proteins as a function of particle size at neutral pH for hydrophobic and positively charged particles.

The reported studies are summarized in Table 3.

**Table 3:** Detailed summary of computational protein corona studies.

**Device material**	**Protein**	**Method**	**Experimental data**	**Outcomes**	**References**
Graphene	Bovine fibrinogen Immunoglobulin Transferrin Bovine serum albumin	MD	Protein adsorption (fluorescence spectroscopy) Atomic Force Microscopy Circular dichroism	Protein affinity with the surface Structural changes	Chong et al., 2015; Gu et al., 2015
Carbon nanotubes	Bovine fibrinogen Immunoglobulin Transferrin Bovine serum albumin	MD	Atomic Force Microscopy Circular dichroism	Protein affinity with the surface Structural changes	Ge et al., 2011; Gu et al., 2015
Gold particles/rods/slabs	β_2_-microglobulin Bovine serum albumin Bovine beta-lactoglobulin Glutathione S-transferase	MD	Circular dichroism X-ray spectroscopy UV spectroscopy Surface plasmon resonance	Structural changes	Wang et al., 2013; Brancolini et al., 2014; Yang et al., 2017; Ma et al., 2018
Gold slab	Aβ_16−22_ peptide	MD + Metadynamics	Sum generation frequency spectroscopy	Structural changes Affinity with the surface	Bellucci et al., 2016
Hydroxyapatite	Bone morphogenetic protein 2	MD	No	Affinity with the surface Structural changes	Dong et al., 2007, 2011
Fullerene	Human serum albumin	MD	Comparison with data from the literature	Binding energies Structural changes	Leonis et al., 2015
Titanium oxide	L–histidine Bone morphogenetic protein 2	MD	Attenuated total reflectance fourier transform infrared spectroscopy	Binding energies Structural changes	Utesch et al., 2011; Mudunkotuwa and Grassian, 2014
Graphite	Bone morphogenetic protein 2	MD	No	Binding energies Structural changes	Utesch et al., 2011
Molybdenum disulfide nanoflakes	K+ channels	MD	Electrophysiology	Binding affinity Consequences on protein functionality as K+ channel	Gu et al., 2018
Functionalized self-assembled monolayers	LKα14 LKβ15	MD + Metadynamics	Comparison with literature	Binding free energies Structural changes	Deighan and Pfaendtner, 2013
Silica surface	GGKGG peptide Chymotrypsin	MD + Metadynamics	Circular dichroism spectra	Binding free energies at different environmental conditions Structural changes	Hildebrand et al., 2018; Prakash et al., 2018
Generic hydrophobic nanoparticle	α_1_-antitrypsin human serum albumin transferrin immunoglobulin G Fibrinogen α_2_-macroglobulin	CG	No	Binding energies Structural changes	Lopez and Lobaskin, 2015
Gold nanoparticles	Insulin Fibrinogen	CG	No	Competitive binding Structural changes	Tavanti et al., 2015; Quan et al., 2017
Silica nanoparticles	Lysozyme	CG (MARTINI)	No	Curvature effects on lysozyme adsorption	Yu and Zhou, 2016
Generic hydrophobic/hydrophilic nanoparticle	Bovine serum albumin	CG (DPD)	No	Binding energy as a function of size and surface characteristics	Ding and Ma, 2014

Although the reported examples of simulations at the CG scale provide interesting findings, they are not coupled with validation against experimental data; therefore, the results should be taken as qualitative theoretical considerations. Notably, the literature offers many examples concerning inorganic nanoparticles (gold, silica) or carbon-based materials (graphene, carbon nanotubes). To our best knowledge, polymeric systems are not widely investigated. This is due to a lack of validated parameters as well as intrinsic issues related to system modeling since, by virtue of their ordered atomic arrangement, inorganic surfaces can be more easily described than a polymer surface composed of a disordered random coil.

To summarize, at this stage, molecular modeling of the protein corona cannot replace experimental activity, and its use as a purely predictive tool is currently premature. This is due, on the one side, to the intrinsic complexity of the system under investigation and, on the other side, to the lack of systematic validation against experimental data. Many examples discussed in the literature are purely theoretical, and only a few recent studies have critically validated simulation outcomes with experiments. In addition, comparison with experimental data is only performed *in vitro*; the complexity of the *in vivo* environment still constitutes an arduous challenge because of the wide range of proteins present in the blood flow and their mutual interactions.

It is important to take into account another limitation of the method: usually, the investigation is focused only on the proteins directly adsorbed on the nanoparticle, usually modeled as a flat surface if the particle size is much larger than that of the protein. Small nanoparticles can be entirely included in the simulations, while in intermediate cases the molecular model of the surface must account for curvature effects.

Molecular simulations must be intended as the ideal complement to experimental activity *in vitro*. As shown in Table 2, simulation outcomes can be compared with the corresponding experimental information, providing a deeper understanding thanks to the detail provided at the molecular level.

The road toward purely predictive simulations is still long and arduous, but the main points to be addressed are clear. On the one side is the development of more reliable force fields that can accurately capture the structural transitions of proteins (in terms of both secondary and tertiary structure) after adsorption. On the other side is a systematic validation of simulations with experimental data, which can clearly highlight the strong and weak points of the molecular model and the computational technique and thus where and how to improve them. The link between experiments and simulations is becoming stronger and tighter, since it is possible to compute theoretical quantities (such as circular dichroism spectra) that can be directly compared with the corresponding experimental outcomes. The validation of full atomistic models is an unavoidable condition for exploiting the main advantages of coarse-grained models, which must be properly parameterized against more accurate simulations at the molecular level in order to obtain robust and reliable results.

### Nanoparticle–Cellular Membrane Interactions

Molecular modeling can also be employed to investigate the interactions of drug molecules and nanocarriers with lipid bilayers that act as a simplified description of the complex and heterogeneous cellular membrane. Full atomistic MD simulations are the method of choice when small drug molecules are involved, while CG models are the only opportunity if the interest lies in bigger entities like polymer nanoparticles. A detailed molecular model of a cellular membrane, which includes several kinds of lipid molecules as well as transmembrane proteins, is still out of reach, although progress has recently been made in this direction (Ingolfsson et al., 2014), as recently reviewed (Ingolfsson et al., 2016; Marrink et al., 2019). This is due not only to the long time scales needed for achieving converged results but also to the lack of the experimental data for complex membranes (that is, composed of different lipid molecules) needed to parameterize and validate molecular models. For this reason, the cellular membrane is usually represented as a homogeneous bilayer (i.e., which contains only one kind of lipid molecule such as dipalmitoylphosphatidylcholine) or a simple heterogeneous membrane (with two different lipid molecules and sometimes cholesterol). In this framework, molecular modeling can be used to qualitatively understand the impact of nanocarrier formulation and the presence of adsorbed proteins on non-specific cellular uptake (that is, not mediated by a receptor).

A typical application of MD simulations is the study of the permeation of drug molecules through lipid bilayers, which mimic cellular membranes. Because of the energy barrier related to membrane crossing, the application of enhanced sampling methods becomes unavoidable. Further post-processing by means of an inhomogeneous solubility–diffusion model allows the evaluation of a position-dependent diffusion coefficient through the lipid bilayer as well as the overall permeation coefficient (Dickson et al., 2017). In another study, Bruno et al. (2015) elucidated the binding mechanism of the multiple sclerosis biomarker CSF-114 peptide to membrane using an unbiased atomistic MD approach inspired by the binding free-energy method, funnel metadynamics (Limongelli et al., 2013).

This approach provides very useful insights, since it allows the relation of the observed permeation of different drug molecules to the specific interactions at the atomic level, such as hydrogen bonds. On the other hand, the use of full atomistic simulations limits the applicability of this analysis to small drug/peptide molecules (up to a few hundreds of Da). The study of nanoparticle permeation with atomic detail would lead to unfeasible or extremely challenging simulations due to the size of the system and the long time scales needed to reach converged results. Because of this, coarse-grained simulations are the method of choice for the study of nanoparticle–cell membrane interactions, as widely discussed in the literature (Rossi and Monticelli, 2014, 2016; Ding and Ma, 2015; Ge and Wang, 2016). For the same reasons, there has been an increase of interest in the use of CG simulations for the study of transmembrane proteins (Lelimousin et al., 2016). In a recent study (Molinaro et al., 2018), a MARTINI model was employed to study the introduction of a membrane protein in biomimetic vesicles (leukosomes) obtained through a microfluidic-based setup. CG simulations allowed the impact of glycosylation, steric hindrance of the protein extracellular domain versus the intracellular domain, and relative to vesicle curvature on protein orientation to be highlighted.

Another limitation is shared by both full atomistic and coarse-grained methods: as has been mentioned, cellular membranes are very heterogeneous environments because of the wide range of lipids involved and the presence of several transmembrane proteins and receptors, and simplified models are needed for affordable simulations. Lipid bilayers made of dioleoylphosphatidylcholine (DOPC) and dipalmitoylphosphatidylcholine (DPPC) are commonly used as cell membrane models thanks to the availability of validated parameters for the force fields. Simulations of bilayers with heterogeneous compositions (that is, composed by many different lipid molecules), which would constitute a more realistic cellular membrane model, are hindered by the lack of experimental data for force field validation (Poger et al., 2016). Transmembrane proteins are not included unless the investigation is focused on the interactions with a specific receptor or on the behavior of such proteins.

In summary, simulations at the molecular level of nanoparticle–cellular membrane interactions are usually performed by means of CG methods and are focused on simplified systems made up of a mimicking lipid bilayer and a small nanoparticle (up to 10–20 nm). The investigation of larger particles, although of potential interest, is still limited by the computational effort required and the difficulty of achieving converged results.

The advantages and disadvantages, for both MD and CG, are summarized in Table 4.

**Table 4:** Advantages and disadvantages for nanoparticle–cellular membrane interactions.

**Advantages**	**Disadvantages**
Availability of validated parameters for the simulation of lipid bilayers	Only homogeneous bilayers can be reliably simulated
Particle–membrane interactions at molecular level	Only CG models can be fruitfully used, because of the size of the system, which is still limited to 10–20 nm nanoparticles
Simulation of membrane-crossing by the naked or functionalized particle	Simulation of the non-specific permeation across a simplified model system The influence of receptors is not taken into account
Protein corona and/or nanoparticle surface modification can be accounted for	Hard corona description is very qualitative and must be validated in a previous step

In general, the comparison with experimental data is more challenging. Simulation of naked and decorated particles (i.e., with surface functionalization and/or a hard protein corona) can highlight the different interactions with the cellular membrane and can be compared with the expected and the experimental cellular uptake. In this framework, simulations are expected to give those insights at molecular resolution, which cannot be obtained experimentally; this reinforces the need to have previously validated models of protein–particle interactions and model lipid bilayers. Computational efforts are currently focused on parametric simulations, where the influence of particle hydrophilicity/hydrophobicity (including charge), coating (e.g., PEGylation), shape, and size on membrane permeation and induced stresses are qualitatively evaluated.

The examples offered by the literature involve generic nanoparticles with different shapes or functionalization (Yang and Ma, 2010; Ding and Ma, 2012, 2014; Li and Hu, 2014; Li et al., 2014), gold nanoparticles (Lin et al., 2010; Rossi and Monticelli, 2016; Salassi et al., 2017; Lunnoo et al., 2019), and polymer systems (Schulz et al., 2012) such as dendrimers (Rossi and Monticelli, 2014, 2016), polystyrene (Rossi and Monticelli, 2016), and polyelectrolytes (Rossi and Monticelli, 2016).

Ding and Ma (2014) employed dissipative particle dynamics to study the influence of human serum albumin corona (*vide supra*) around hydrophobic or positively charged nanoparticles on membrane permeation. They found that at physiological pH, the HSA corona promotes particle adhesion on a DPPC lipid bilayer model of a cell membrane thanks to the specific interactions with the protein coating of a 3-nm hydrophobic particle. They also investigated the impact of pH and membrane charge.

Li et al. (2014) studied through a coarse-grained model and dissipative particle dynamics the effect of PEG grafting density (0.2–1.6 chains nm^−2^) and molecular weight (550–5000 Da) on the internalization of an 8-nm particle, proposing a optimal choice of parameters for maximizing cellular uptake. They also characterized the uptake process in detail, identifying three stages: membrane bending (0 < t < 122 ns), membrane monolayer protruding (122 < t < 750 ns), and equilibrium (t > 750 ns).

Recently, Lunnoo et al. (2019) employed the MARTINI CG model to simulate the cellular uptake of gold nanoparticles. Notably, they employed a more complex mammalian cell model previously proposed by Ingolfsson et al. (2014), which includes 63 different lipid species asymmetrically distributed in the bilayer. This allowed the limitations of simple models to be overcome and the complexity of a more realistic cellular membrane to be accounted for; indeed, they found that neutral 10-nm nanoparticles experienced an endocytic pathway with a DSPC/DSPG model membrane, while they exhibited direct translocation across the more complex model of a mammalian membrane. They also characterized the energy barrier related to membrane crossing by changing the shape and charge density, also taking particle aggregation into account.

Similarly to protein corona simulations, in this framework, molecular modeling must still be considered as a complementary tool to experimental activity and not as an alternative. Although it provides interesting insights, the lack of systematic experimental validation hinders the application of molecular simulations as a predictive tool. It is also necessary to take into account the inherent approximations of coarse-grained models, where some kinds of interactions are poorly accounted for. In addition, there are still some limitations concerning the size of the device; according to examples in the literature, the maximum investigated nanoparticle size is about 20 nm. Simulations of larger devices not only increase the number of beads but also require very long calculations to achieve converged results: the required computational effort is not always feasible. This issue could in principle be overcome by employing, e.g., implicit solvent methods, which further improve computational efficiency by representing the solvent as a continuum (and thus reducing the number of explicit beads in the system) at the price of an additional approximation. The implicit solvent parameterization of the MARTINI force field, called Dry MARTINI, is currently validated only for lipid bilayers, although it has been shown that it can also be used for polymeric systems after a proper re-parameterization (Bochicchio and Pavan, 2017). In general, the use of implicit solvent methods requires an accurate parameterization and validation with experimental data or more detailed simulations at an atomic scale. Currently, only qualitative insights concerning more realistic systems (in terms of particle size) can be obtained through the simulation of smaller devices.

## Conclusions and perspectives

Simulations at the molecular level, despite the discussed limitations and drawbacks, constitute a powerful tool for improving understanding of the governing phenomena at the nano–bio interface. The intrinsic peculiarities of molecular modeling, which account for the synergistic effects of several factors (particle material, protein adsorption, environmental effects, interactions with cellular membranes, *et cetera*), can provide some insights that are challenging or impossible obtain experimentally, thanks to the molecular resolution. The increasing availability of computational resources, the development of improved force fields (that are more accurate), algorithm optimization, and theoretical advancements are constantly pushing molecular simulations beyond their limits, slowly overcoming the current issues.

Focusing on the protein corona, the *conditio sine qua non* for a meaningful simulation is a validated force field, which allows a reasonable description of secondary and tertiary structures to be obtained and a robust sampling of the most relevant conformation. Indeed, discrepancies in the description of protein structural transitions inevitably affect result reliability and the subsequent steps (e.g., the study of the interaction of a protein-decorated particle with a cellular membrane). Descriptive capabilities are known *a priori*, since they are addressed in detail in several papers and FF reference papers. The development and the improvement of force fields (not only for proteins) are always ongoing, and updates are periodically released and discussed in the scientific literature. This refinement process is currently taking advantage of new state-of-the-art techniques such as machine learning (Debiec et al., 2016).

Molecular dynamics simulations provide detail at an atomic level, but they are limited by the time scale of many phenomena of interest (such as protein folding/unfolding, slow binding/unbinding kinetics), which is beyond that accessible through standard simulations. The development of enhanced sampling methods allows this issue to be alleviated and allows a more comprehensive ensemble of conformations to be obtained. Currently, the extensive application of such methods is still hindered by the size of the molecules under investigation, which cannot exceed, in the case of proteins, a few tens of amino acids in order to obtain reliable and converged results. Further improvements of the method itself and optimization of computational protocols and algorithms could allow the investigation to be focused on larger and more complex proteins.

Coarse-grained models, along with suitable methods to study system dynamics, have emerged as an attractive choice when molecular dynamics simulations are unfeasible because of the time and length scales involved. Indeed, despite the loss of atomic detail, CG models have proved that the fundamental physical/chemical peculiarities lie at the molecular model. However, in order to obtain reliable results, careful parameterization and validation against experimental data still represent essential steps that are not always addressed.

Simulations are mainly focused on inorganic particles (gold, silica) or carbon-based devices (graphene, carbon nanotubes), while there are no relevant examples concerning polymer nanoparticles. This can be attributed to the fact that molecular models of inorganic particles are easier to build given the availability of reliable force field parameters together with their known and well-parametrized structural properties.

Many efforts are also being devoted to the development of more realistic models of cellular membranes, as recently reviewed (Ingolfsson et al., 2016; Marrink et al., 2019). This aspect cannot be underestimated, because the reliability of the results concerning drug or nanocarrier–cell membrane interactions of course requires a robust description of a cell membrane with a suitable level of approximation.

The available force fields provide validated parameters for small sets of lipid molecules (although the number of available compounds increases in every FF update), and it is difficult to validate simulations of heterogeneous membranes (that is, made up of several kinds of different lipid molecules) because of the lack of suitable experimental data. In this regard, a first attempt has been performed by Ingolfsson et al. (2014), who employed a CG MARTINI model to simulate an idealized mammalian plasma membrane, including more than 63 lipid species asymmetrically distributed in the bilayer. Marrink et al. (2019) recently published a comprehensive review that summarizes all the advancements in the field and clearly describes the ultimate goal for comprehensive modeling: the simulation of a membrane with hundreds of different lipids, with a large variety of transmembrane as well as peripherally bound proteins and realistic gradients of metabolites, ions, and pH. Although this “definitive” simulation is still far off, there are in the literature some interesting attempts to model complex systems, such as viral envelopes and mesoscale simulations remodeling eukaryotic cell membranes (Marrink et al., 2019).

In conclusion, simulations at the molecular scale have emerged as a fruitful tool to complement the insights provided by experimental activity and obtain a deeper understanding of the main phenomena behind the observed behavior. Despite their use becoming more and more widespread, there are still some points that need to be addressed in the near future to overcome the current limitations:

- Extensive application of plain and enhanced sampling simulative methods to study the conformational changes of the most abundant plasma proteins;
- Availability of force fields of increased accuracy;
- Extension of the study of protein–particle interactions to polymeric systems prone to bind to the NP surface;
- Systematic and rational validation of molecular models with *ad hoc* experimental data;
- Extensive validation of CG models for nanoparticle–cellular membrane interactions;
- More realistic models of cellular membranes.

## Author Contributions

TC performed the literature research and wrote the first draft of the paper. All authors discussed and approved the contents of the manuscript and contributed to the final version by reading and editing.

### Conflict of Interest

The authors declare that the research was conducted in the absence of any commercial or financial relationships that could be construed as a potential conflict of interest.
